# On Italian neuropathology and its decline: when and how will it be revived?

**DOI:** 10.1007/s10072-025-08008-x

**Published:** 2025-02-08

**Authors:** Orso Bugiani, Marianna Bugiani

**Affiliations:** 1 Fondazione IRCCS Istituto Neurologico Carlo Besta, Milano, Italy; 2 Department of Pathology, Amsterdam University Medical Center, Amsterdam, The Netherlands

In Italy, neuropathology, i.e. the study of changes in nervous tissue and how they occur and lead to dysfunction [1], has long since lost its academic status and role in health care. The purpose of this note is to report the events that may explain some of the dynamics of this failure.

**Fig. 1:**
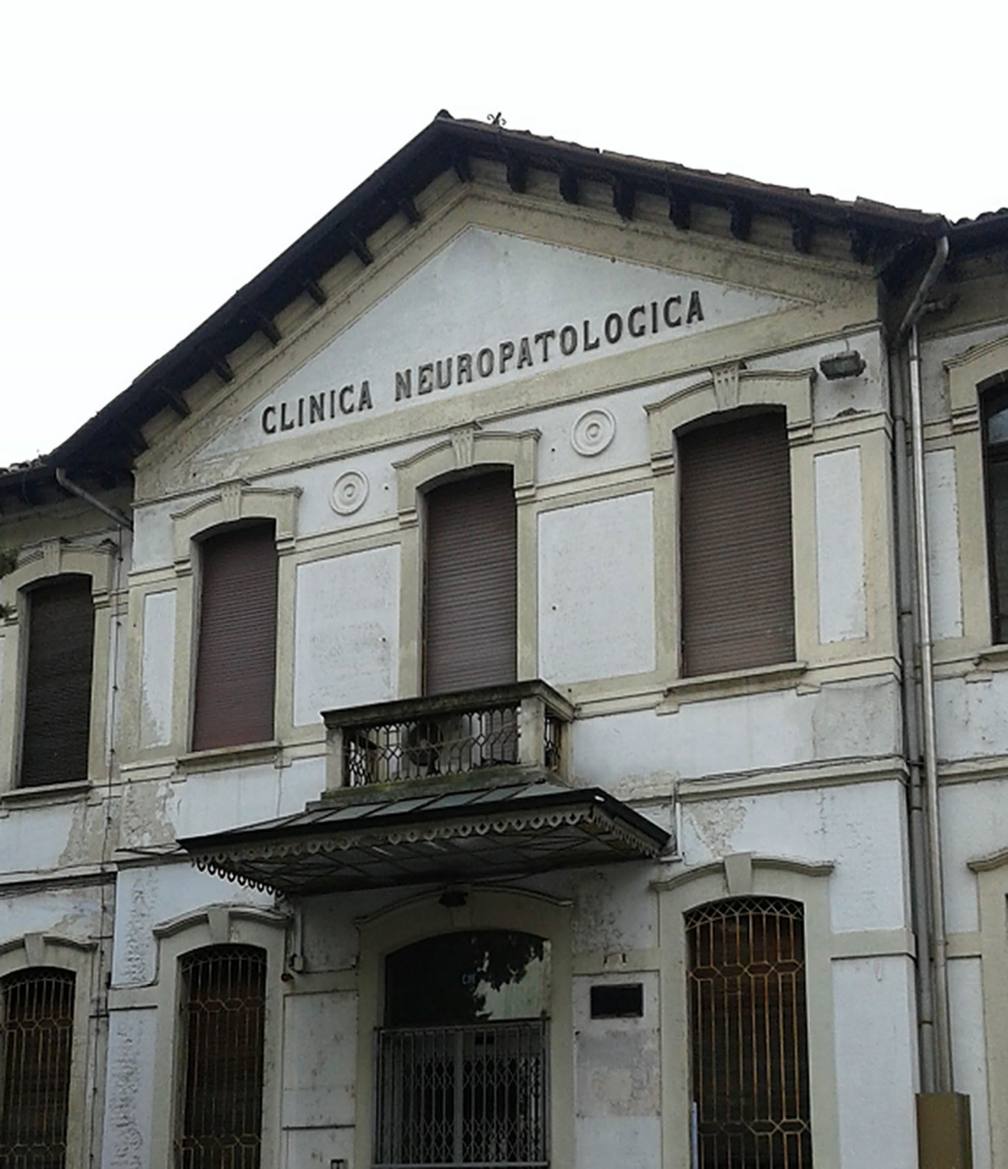
The front of the Clinica Neuropatologica of the University of Pavia, founded in 1914 by Casimiro Mondino for the study of nervous diseases

The term “neuropathology”, introduced in the late 1700s, has often referred to the clinical pathology of the nervous system, anticipating the term “neurology” since the mid-1800s. Under a semantic chronology, this may illustrate the evolution of phrenology, as the study of the mind, into neurology, as the study of the brain, which emerged following the growth of disciplines such as physiology, pathology, and the pathological and comparative anatomy of the brain. According to the phrenologist Miraglia (1814–1885), these disciplines would have made it possible to study the dysfunctions of “the organ that gives the mind the power to develop and manifest its faculties” [2], leading to the conceptual separation of the brain from the mind. This prediction was confirmed in 1907 when Golgi, Lombroso, Mingazzini, Tanzi and their students founded the *Società Italiana di Neurologia* (SIN), the successor of Miraglia’s *Società Frenopatica*. The new name prevailed over the rival* Società Freniatrica e Neuropatologica* [2], suggesting that most of the founders were aware of the distinction between neurology and neuropathology in the modern sense of the term. Nevertheless, neuropathology and neurology have been synonymous at least since 1883, when the first chair of neuropathology was created in Rome for the neurologist Sciamanna. This ambivalence may be the reason why some neurological institutes were designated as neuropathology clinics (Fig. 1) and many neurologists were registered as neuropathologists at early SIN meetings. The same ambiguity affected several journals, including the *Giornale di Neuropatologia* (founded in 1882) and the *Rivista Italiana di Neuropatologia, Psichiatria ed Elettroterapia* (1907). To ascertain a definitive stance on this matter, it is necessary to wait for the issue of *Neurologica: Rivista Italiana di Neuropatologia e Psichiatria* in 1924, which became *Annali di Nevrologia* in 1927. In the 1930s, the name was officially changed when the academic course “Neuropathology and Psychiatry” became “Nervous and Mental Diseases”. Nevertheless, in 1980, the academic curriculum of the Dental School once again designated neurology as neuropathology.

**Fig. 2:**
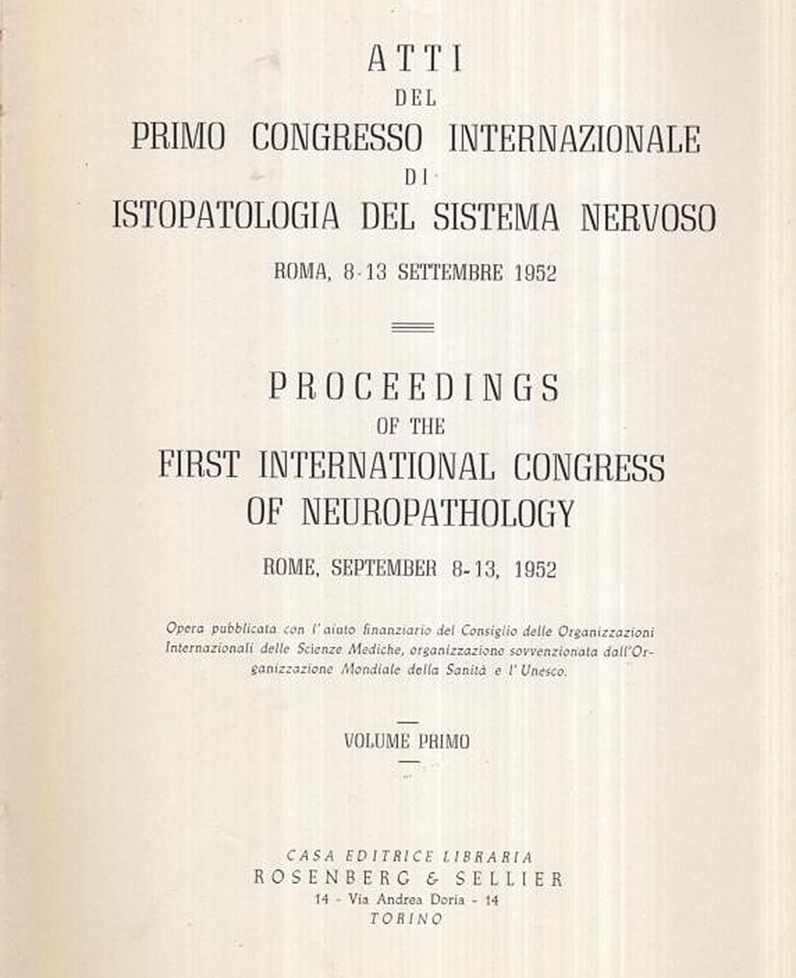
Title page of the Proceedings of the First International Congress of Neuropathology, Rome 1952. In the Italian version, “neuropathology” was changed to “histopathology of the nervous system”, perhaps as a result of a doctrinal dispute over the academic affiliation of the discipline involving the organizers of the congress

**Fig. 3:**
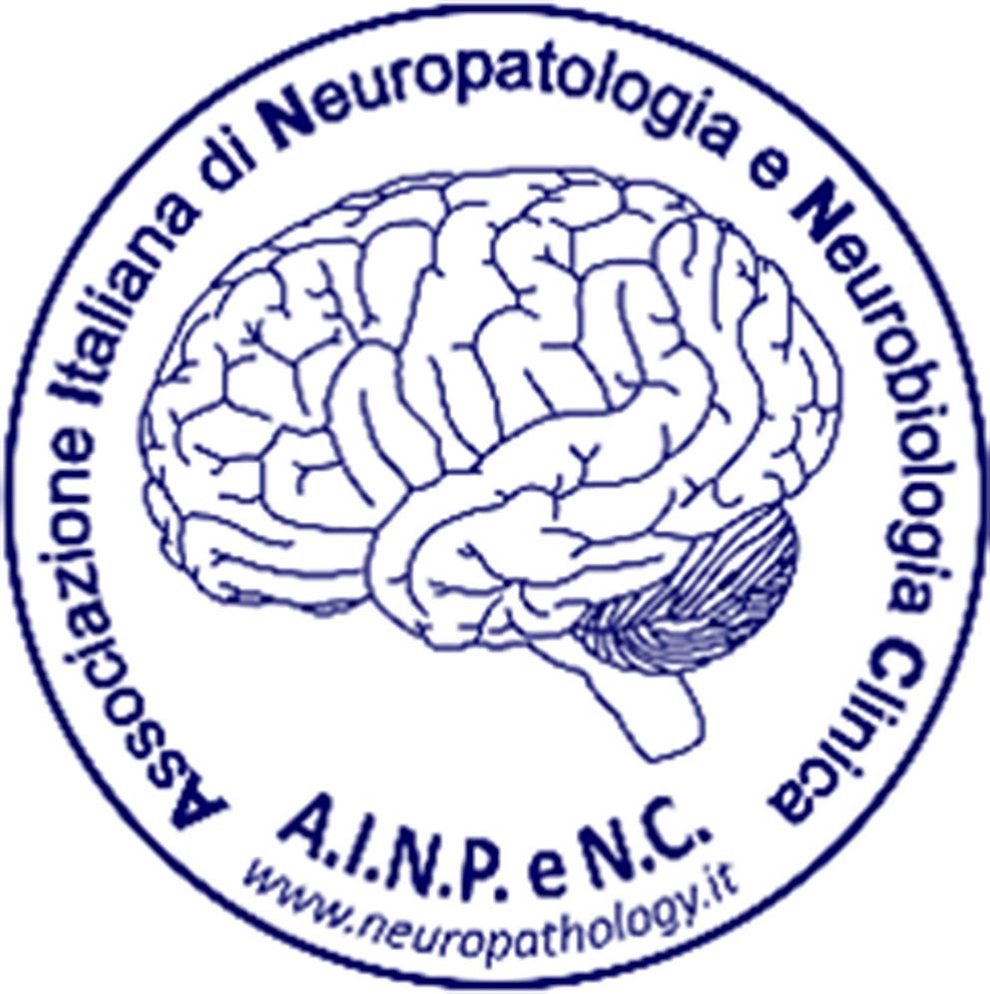
Logo of the Italian neuropathologists

In contrast to the academy, “neuropathology” was used in psychiatric hospitals only for the study of diseased brain tissue. Since the late 1800s, this study has been successfully practiced in these hospitals by neurologists, psychiatrists, and pathologists (including Alzheimer’s pupils Bonfiglio, Cerletti and Perusini). However, its importance declined after World War I when psychiatric research shifted its focus to investigate social factors contributing to mental distress. Gozzano, professor of neurology and psychiatry in Rome, focused on this decline when, as president of the 1952 Congress of the International Society of Neuropathology (Fig. 2), he told his audience that neuropathology was nearing its end. However, it was soon revived by the introduction of neurochemistry, histochemistry and electron microscopy. In Italy, neurologists were the most active participants in this resurgence, also responding to the call of the International Society by establishing the *Sezione di Neuropatologia* (affiliated with the SIN) in 1965. This initiative has been successful in attracting neuroscientists from diverse backgrounds and in gaining recognition for teaching neuropathology along with neurology at the university level [3]. The affiliation of neuropathology with neurology suggests that a forward-looking agreement may have been reached between pathologists and neurologists based on respect for individual expertise in neuropathology. Later, however, the teaching of neuropathology was reassigned to pathologists under the name ”pathology of the nervous system”. Unfortunately, regardless of its academic affiliation, neuropathology’s training and practice have not met the expected standards of success. Furthermore, the emergence of ancillary disciplines (mainly muscle and peripheral nerve pathology, and brain tumors) prompted the formation of autonomous associations that absorbed interest and members, reducing the status of the *Sezione* to a club focused on neurodegeneration, known as the *Associazione Italiana di Neuropatologia e Neurobiologia Clinica* (Fig. 3).

One consequence of the ongoing ambiguities and changes in this field has been that many neuroscientists have relocated abroad to pursue their neuropathological ambition. The first to emigrate was Armando Ferraro, a psychiatrist who, in the 1950s, became editor-in-chief of the journal of the American Association of Neuropathologists. In recent years, the decision to move abroad has sometimes been influenced by the lack of suitable academic positions in Italy. The movement of neuropathology out of neurology has not yet created new positions. Perhaps this move has improved diagnostics and research in neuro-oncology, but it has also reduced the number of laboratories active in neurological institutions. Furthermore, the number of autopsy-based brain tissue studies has declined, as has interest in the discipline and its clinical-anatomical implications. Ultimately, this may raise the question of what currently constitutes Italian neuropathology.

Since 1994, the European Confederation of Neuropathological Societies (Euro-CNS) has been actively promoting neuropathology, as many textbooks have done for a century, demonstrating that the discipline “obviously requisite to an understanding of neurology, neurosurgery and psychiatry”, as Adams and Sidman put it in their 1968 *Introduction to Neuropathology*. We should add clinical and laboratory disciplines (including neurobiology and neurogenetics, neuroimaging and positron emission tomography, brain-banking and artificial intelligence-assisted procedures) that explain and are explained by neuropathology. This makes neuropathology a discipline that applies knowledge from different sources to the study of the nervous tissue when this is the target of disease [1]. Thus, from a heuristic perspective, it can be seen as a place where the history, methods, data, and hypotheses about lesions and dysfunctions of the nervous system are brought together to help neuroscientists develop their ideas. It is also important to note that the scientific weight of neuropathology is likely to increase as more biological and clinical knowledge is discovered. For these reasons, it would be useful to finally give Italian neuropathology the academic status of an autonomous discipline bridging neuroscience and pathology, as a first step to revitalize it for the benefit of neurology and psychiatry.
